# Recent trends in machine learning and deep learning-based prediction of G-protein coupled receptor-ligand binding affinities

**DOI:** 10.3389/fbinf.2025.1712577

**Published:** 2026-01-12

**Authors:** Joshua Stephenson, Konda Reddy Karnati

**Affiliations:** Department of Natural Sciences, Bowie State University, Bowie, MD, United States

**Keywords:** drug–target binding affinity, G protein–coupled receptors (GPCRs), binding affinity prediction, machine learning (ML), deep learning (DL)

## Abstract

Accurately predicting protein-ligand binding affinity is key in drug discovery. Machine Learning and Deep Learning methods used in the drug discovery process have advanced the prediction of drug–target binding affinities, particularly for G protein–coupled receptors (GPCRs), a pharmacologically significant yet structurally heterogeneous protein family. In this review, binding affinity prediction models are examined and organized according to sequence-based one-dimensional, graph-based two-dimensional, and structure-based three-dimensional frameworks. Sequence-based models utilize convolutional neural networks for high-throughput screening. Recently published models incorporated attention mechanisms and self-supervised learning, enhancing interpretability and reducing dependence on annotated datasets. Graph-based models employ graph neural networks and molecular contact maps to capture topological features, enabling substructure-sensitive predictions. Structure-based approaches integrate spatial and conformational data into high-resolution interaction models. The hybrid use of these three approaches could significantly increase the success rate of *in silico* models for drug discovery, particularly for GPCRs.

## Introduction

Binding affinity is the key parameter in drug discovery for predicting the strength between protein and ligand (Spassov, 2024; Gilson and Zhou, 2007). Predicting accurate binding affinity is challenging with current computational methods; strategies such as molecular docking are used for binding affinity prediction, but do not yield highly satisfactory results (Spassov, 2024). To overcome this limitation, binding affinity prediction models using Machine Learning (ML) and Deep Learning (DL) have become more prevalent in the drug discovery workflow. These models assist with the estimation of the strength of interactions between small molecules and biological macromolecules, which are often calculated as K_d_, K_i_, or IC_50_; this guides prioritization of compounds before costly experimental assays. The dimensionality of the input representations, one-dimensional (1D), two-dimensional (2D), and three-dimensional (3D) models, can broadly rank the models. 1D models operate on sequences or chemical strings, 2D models use graph-based molecular topologies or contact maps, and 3D models use inputs of spatial coordinates of atoms or coarse-grained conformations (Chen et al., 2016; Wang, 2024; Nguyen et al., 2024; Wang et al., 2015).

**FIGURE 1:**
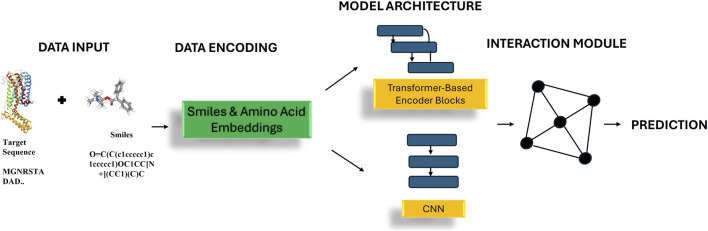
Workflow of ML/DL based binding-affinity prediction, beginning with data input and encoding, proceeding through the model architecture and the interaction module (where SMILES and amino-acid embeddings are fused), concluding with the affinity prediction.

DL, which is a subset of ML, is a branch of artificial intelligence (AI) that enables computers to observe patterns from data and make predictions without strict rule-based programming (LeCun et al., 2015; Mahesh, 2020). Traditional ML approaches were lacking in their ability to process original data in its base state; they relied on engineered descriptors and algorithms such as decision trees, support vector machines, or random forests (Mahesh, 2020; LeCun et al., 2015). In contrast, DL leverages layered neural network architectures to change internal parameters by using backpropagation within the algorithm (Figure 1) (LeCun et al., 2015). Over the last decade, these methods have transformed early-stage drug discovery by accelerating virtual screening and reducing the difficulties of synthesis experiments (Paul et al., 2021; Blanco-González et al., 2023). They have enabled structure-based virtual screening (Cheng et al., 2012; Kitchen et al., 2004), kinase selectivity profiling (Davis et al., 2011), and the identification of ligand-binding residues (Chen et al., 2014).

G-protein-coupled receptors (GPCRs) constitute the most prominent family of druggable membrane proteins and control multiple downstream cellular signals. Despite accounting for one-third of marketed therapeutics, many GPCRs lack effective pharmacological treatments (Hauser et al., 2017). Cryo-EM and X-ray crystallography advances have improved the use of high-resolution GPCR structures (Congreve et al., 2020), revealing conserved activation motifs (Venkatakrishnan et al., 2013) and enabling structure-based design. Although recent cryo-EM structures have improved coverage of GPCRs, high-resolution structures capturing receptor activation states and receptor conformations stabilized by a particular ligand remain uncommon, especially at allosteric sites; this complicates the accuracy of binding affinity inference and subtype selectivity (Congreve et al., 2020; Wacker et al., 2017; Krishna Kumar et al., 2019; Xia et al., 2021). However, predicting GPCR-ligand binding affinity can be difficult since the membrane receptors are dynamic and can adapt multiple conformations, while their endogenous peptide ligands vary widely in sequence, length, and post-translational modifications; together, these limit available structural data and make it hard for models to generalize beyond training sets. Due to GPCRs having multiple allosteric and orthosteric sites with ligand-specific pocket arrangements, any fixed representation risks missing relevant details (Latorraca et al., 2017; Christopoulos, 2014). Benchmarking also shows that distinguishing true binders from closely related decoys is difficult due to binding-pocket similarity across receptors (Hoegen Dijkhof et al., 2025). As a result of these features, the use of 1D sequence-only models, 2D graph-based models, 3D models, and structure-based models can potentially improve the accuracy of binding affinity predictions and lower the cost of resources for both *in silico* and experimental processes.

## 1D binding affinity models

**FIGURE 2:**
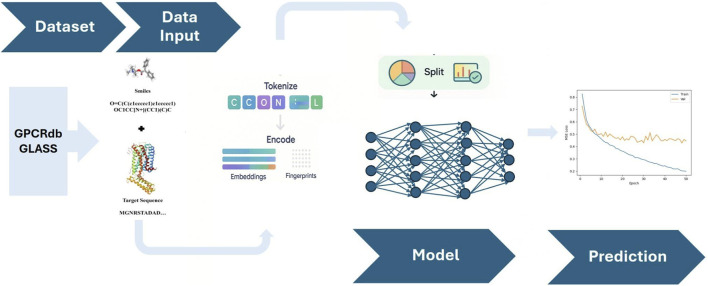
1D binding affinity models utilize linear representations of molecules (e.g., SMILES) and proteins (amino acid sequences) processed by CNNs or recurrent layers to predict interactions without requiring structural data (Öztürk et al., 2018). The image above illustrates a 1-D Binding Affinity Prediction model.

1D ML models process sequential data, typically in 1D formats such as text, time series, or biological sequences, to extract patterns and make predictions (Kiranyaz et al., 2021). Proteins as amino-acid sequences and ligands encoded as canonical SMILES (Simplified Molecular Input Line Entry System) are fed into tokenized sequences within convolutional or recurrent networks. The use of 1D sequence-based models enables rapid, high-throughput screening due to not having to rely on structural data (Öztürk et al., 2018; Wang, 2024). Their Convolutional Neural Networks (CNN) encoders have given competitive results (Table 1.) on benchmark datasets (e.g., Davis, KIBA) and can outperform classical docking in some instances, making them efficient and easy to scale (Öztürk et al., 2018; Öztürk et al., 2019; Kitchen et al., 2004). Recent self-supervised approaches further boost their effectiveness by learning useful representations from extensive unlabeled data, reducing dependence on labeled data (Schuh et al., 2025; LeCun et al., 2015). 1D encodings can also capture pharmacological properties in multi-task setups, which broadens their role as initial filters (Brahma et al., 2025; Jabeen and Ranganathan, 2019). Recent studies have shown that coupling 1D CNN encoders for SMILES and protein sequences (Figure 2), followed by fully connected layers to regress binding affinity, could produce worthwhile results (Öztürk et al., 2018; LeCun et al., 1998).

**TABLE 1:** The table below shows the metrics like CI, MSE and RMSE of various models.

Model	Dataset	CI	MSE	RMSE
DeepDTA	KIBA	0.863	0.194	0.440
DeepDTA	Davis	0.878	0.261	0.51
WideDTA	KIBA	0.875	0.179	0.423
WideDTA	Davis	0.886	0.262	0.511
DeepAffinity	Davis/KIBA	N/A	N/A	N/A
GSAML-DTA	Davis	0.896	0.201	N/A
AiGPro	36 GPCRs (per-receptor)		0.09–3.15	N/A
DEAttentionDTA	CASF-2016 (core)	0.82	N/A	1.224

One of the models, DeepDTA (Öztürk et al., 2018), incorporated this approach and, despite its simplicity, outperformed classical docking on the Davis and KIBA datasets and has the potential to be adapted for GPCR-centric datasets. Its variant, WideDTA, expands on this by incorporating additional textual descriptors and interaction contexts (Öztürk et al., 2019); all recent 1D models' description is listed in Table 2. Barlow Twins, a self-supervised architecture, was introduced to learn embeddings from extensive unlabeled data, achieving optimal performance on diverse drug target interaction (DTI) sets while requiring fewer labeled examples (Schuh et al., 2025). Within GPCR drug discovery, multi-task sequence models such as AiGPro can classify both agonism versus antagonism across receptor subfamilies concurrently, which demonstrates how 1D encodings can capture pharmacological structures in addition to affinity (Brahma et al., 2025). These models are supported by trends in ligand discovery using ML-based algorithms (Jabeen & Ranganathan et al., 2019; Blanco-González et al., 2023; Lorente et al., 2025; Öztürk et al., 2018).

**TABLE 2:** This table combines the comparisons across 1D, 2D, and 3D models, showing the strengths and weaknesses of each model as well as their architecture and metrics suited to each task. For the 1D models, the Concordance Index (CI), which measures how well predicted binding affinities preserve the rank ordering of experimental values, is used; CI > 0.85 is generally considered strong (Öztürk et al., 2018). For 2D models, Root Mean Squared Error (RMSE) is used, which is a standard regression metric where lower values indicate more accurate predictions of continuous affinity readouts, which is widely used on benchmarks like KIBA and Davis (Öztürk et al., 2018). 3D models incorporate spatial and conformational data, which could entail the fusing of ligand graphs, protein sequences, and 3D pocket descriptors (Cai et al., 2022).

Dimension	Model	Architecture	Strengths	Weaknesses	References
1D	DeepDTA	Dual CNN encoders on SMILES and protein sequences	Simple, fast, suitable for high-throughput screening	No explicit structural context; prone to dataset bias	Öztürk et al. (2018)
1D	WideDTA	CNN encoders with enriched token sets for SMILES and proteins	Improved expressivity and slight CI gains over DeepDTA	Sequence-only	Öztürk et al. (2019)
1D	DeepAffinity	Hybrid CNN + BiLSTM encoders; unified sequence model	Captures long-range dependencies; interpretable sequence attention	Heavier computational footprint vs. pure CNNs	Karimi et al. (2019)
1D	Barlow twins DNN (DTI)	Self-supervised; siamese-style encoders for 1D inputs	Leverages unlabeled data; good performance with fewer labeled examples	Still lacks structural context; may inherit dataset biases	Schuh et al. (2025)
1D	AiGPro	Multi-task sequence model profiling GPCR agonism vs. antagonism	Captures pharmacological outcomes beyond affinity; cross-receptor generalization	Sequence-only inputs	Brahma et al. (2025)
2D	DEAttentionDTA	Dynamic embedding + self-attention on compound/protein features; GNN/CNN hybrids	Improved RMSE/R^2^ on BindingDB; interpretable attention over substructures/residues	2D graphs/contact maps ignore stereochemistry; sensitive to noisy inputs	Chen et al. (2024)
2D	GSAML-DTA	Graph neural networks + self-attention with mutual-information regularization	Interpretable attention maps; competitive on KIBA/Davis	Approximate spatial reasoning; limited explicit 3D context	Liao et al. (2022)
3D	DeepREAL	Ligand graphs + protein sequence + 3D pocket descriptors; multi-scale fusion	OOD-robust GPCR activity prediction; AUC >0.85 on challenging splits	Depends on accurate pocket/structure data; higher computational cost	Cai et al. (2022)
3D	GPCR conformational classifier	Supervised ML on MD-derived conformational descriptors	Able to determine active or inactive states (∼87% accuracy reported)	Not a direct affinity model; reliant on MD data quality	Buyanov and Popov (2024)

## 2D binding affinity models

**FIGURE 3:**
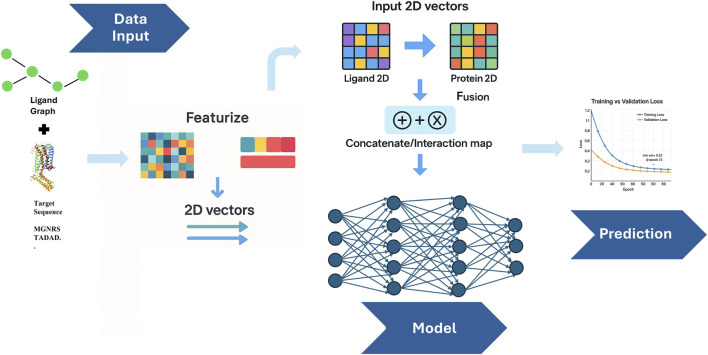
2D binding affinity models represent molecules as graphs where atoms are nodes and bonds are edges, and apply GNNs to capture topological and substructural information (Liao et al., 2022). The image above illustrates a 2-D Binding Affinity Prediction model.

2D models process spatial data represented in two dimensions, such as images, matrices, or other grid-like structures (Figure 3). These models, particularly within DL, are usually built on CNNs, which apply 2D filters to detect local patterns that extract features such as edges, textures, and shapes (Li et al., 2022). 2D models adapt chemical representation to graph structures in which atoms are nodes and bonds are edges, or to residue–residue contact maps for proteins. Graph neural networks (GNNs) can generate annotations along these edges, capturing the local topology and functional group context; this creates a balance between its ability to recognize the complex relationships within a chemical environment and computational effectiveness (Chen et al., 2016; El-Atawneh and Goldblum, 2024). Due to the efficiency of these models, millions of compounds can be screened before 3D docking or simulation while preserving chemical diversity (Sadybekov and Katritch, 2023; Chen et al., 2016).

The model DEAttentionDTA (See Table 2) integrates dynamic embedding with self-attention layers to re-weight atom and residue contributions, significantly improving K_i_ prediction on BindingDB and demonstrating strong similarities to GPCR sets (Chen et al., 2024). GSAML-DTA combines a GNN encoder with self-attention mechanisms and mutual-information shrinkage, yielding interpretable attention maps highlighting substructures while maintaining competitive performance (Liao et al., 2022). 2D models are frequently used to sort millions of compounds before structure-based docking, significantly reducing the pool of molecules while retaining chemically diverse ones. (Chen et al., 2016; Sadybekov et al., 2023; Karimi et al., 2019).

## 3D binding affinity models

**FIGURE 4:**
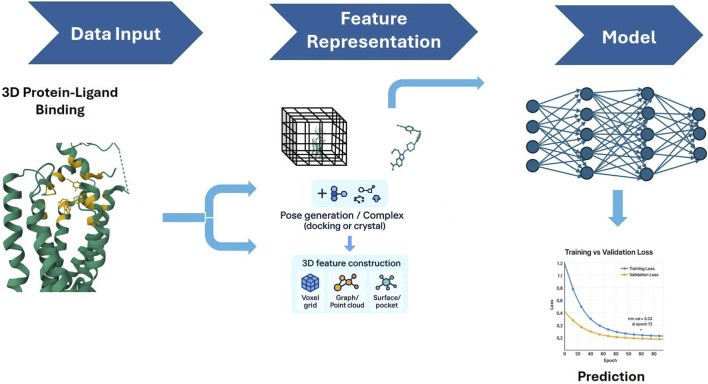
3D binding affinity models incorporate spatial and conformational information using atomic coordinates and pocket structures to simulate molecular interactions with high resolution (Cai et al., 2022). The image above illustrates a 3-D Binding Affinity Prediction model.

3D models (see Figure 4) leverage spatial structural information to capture complex molecular interactions. 3D models introduce spatial coordinates and conformational ensembles, which directly model non-covalent interactions such as hydrogen bonding, π-stacking, and steric clashes. Techniques range from voxelated CNNs to SE (3) equivariant GNNs that respect rotational symmetry. By encoding ligand–target contact patterns as interaction fingerprints, these methods support computational simulations of biological changes and data-driven drug repurposing (Wacker et al., 2017; El-Atawneh and Goldblum, 2024). By leveraging receptor dynamics, these models can distinguish between active and inactive GPCR receptor conformational states, which would improve the quality of ligand design (Buyanov and Popov, 2024).

Studies into this field have resulted in DeepREAL (See Table 2), a model that employs a multi-scale framework that fuses ligand graphs, receptor sequences, and coarse-grained 3D pocket descriptors. Trained not only for accuracy on training distribution, but also to generalize data drawn from different distributions, such as scaffold-split Distributionally Robust Optimization; it effectively predicts GPCR activity for novel chemical profiles (Cai et al., 2022). ML classifiers of GPCR conformational states use structural descriptors to distinguish between inactive, active, and intermediate poses obtained from molecular dynamics (Buyanov and Popov, 2024). Additionally, recurrent neural networks were used to forecast conformational transitions in molecular dynamics simulations (López-Correa et al., 2023). The CB1–Gi complex, which is a high-resolution cryo-electron microscopy (cryo-EM) structure of the cannabinoid receptor 1 (CB1) in complex with the Gi protein (Krishna Kumar et al., 2019), further enables transfer learning where pre-trained 3D encoders are based on ligand-specific affinity labels, bridging experimental structural biology and computational predictions (Xia et al., 2021).

## Discussion

The growing integration of ML and DL in drug discovery has given rise to several binding affinity prediction models, each showing a unique perspective on GPCR-targeted research. These models utilize a specific metric based on the dimensionality being used, as shown in Table 1 above; however, due to these differences in their select metrics, it's difficult to draw a comparison between the models; this highlights the importance of interchangeable splits for comparison analysis and consistent data standards. 1D models such as DeepDTA and WideDTA use sequence-based representations that produce high-throughput virtual screening (Öztürk et al., 2018; Öztürk et al., 2019). DEAttentionDTA and Barlow Twins models use attention mechanisms and self-supervised learning techniques to improve performance while reducing dependence on labeled data (Chen et al., 2024; Schuh et al., 2025). Models such as AiGPro include pharmacological properties such as receptor agonism and antagonism, which go beyond the scope of just adhering to binding affinity (Brahma et al., 2025). However, the limitations of 1D models are in their lack of spatial and structural information, which is crucial for modeling conformational dynamics and ligand-specific binding data (Hauser et al., 2017; Wacker et al., 2017). To provide a solution for this issue, 2D and 3D models introduce greater structural awareness; 2D models like GSAML-DTA and DEAttentionDTA utilize GNNs and self-attention mechanisms to capture local chemical context and observe key substructural features, which can improve functionality and affinity prediction (Liao et al., 2022; Chen et al., 2024). 3D models such as DeepREAL and GPCR Conformational Classifier incorporate spatial coordinates and molecular dynamics conformations that allow accurate modeling of complex GPCR-ligand interactions and receptor activation (Cai et al., 2022; Buyanov and Popov, 2024). Although these models need greater computational resources to operate proficiently, receiving high-quality structural input provides important insight into receptor signaling and potential effectiveness of compounds (Congreve et al., 2020; Krishna Kumar et al., 2019). and molecular dynamics conformations that allow accurate modeling of complex GPCR-ligand interactions and receptor activation (Cai et al., 2022; Buyanov and Popov, 2024). Although these models need greater computational resources to operate proficiently, receiving high-quality structural input provides important insight into receptor signaling and potential effectiveness of compounds (Congreve et al., 2020; Krishna Kumar et al., 2019).

Improving data quality and standardization will be critical in overcoming limitations within this area of research. Inconsistent assay protocols, mixed affinity metrics (K_d_, K_i_, IC_50_), and benchmark biases can distort true generalization and inflate reported performance; adopting consistency between data standards and transparent data workflow reporting are steps that can be used for further advancement within this area. Random splits can leak closely related scaffolds across training and test sets, overestimating performance; scaffold splits provide a stricter estimate of practical generalization to novel chemotypes (Yang et al., 2019). To ensure reliability within these pipelines, incorporate explainable AI during validation, and report results under a scaffold, time, or cluster splits so explanations correspond to stable interactions rather than dataset effects (Ong et al., 2023; Davis et al., 2011). For GPCRs, resources that integrate sequence, structure, and function can benefit reliable cross-study comparisons; clarifying and extending the effective space can propel future innovation of these models. Evaluations should be conditioned on the pocket and receptor state instead of dataset-driven ones to assess the stability of the core framework and chemical changes. The use of databases such as GPCRdb, which offer sequences and structures that can be integrated within docking or structure-based workflows, and GLASS, which provides curated GPCR–ligand pairs and receptor subtype labels that can be useful for training and or validation and scaffold- or time-split tests (Munk et al., 2016; Chan et al., 2015; Hauser et al., 2017; Jabeen and Ranganathan, 2019; Congreve et al., 2020; Nguyen et al., 2024).

By aligning ESM sequence embeddings with pocket alignment features on GPCRdb structures and GLASS subtype labels, multimodal transfer learning can enhance out-of-distribution (OOD) robustness while maintaining clear explanations of the binding mechanism. (Munk et al., 2016; Chan et al., 2015; Lin et al., 2023; Stärk et al., 2022; Corso et al., 2022). The difficulty posed by screening multi-billion compound libraries would suggest that ML-guided pre-screening will remain a practical path; however, narrowing candidates before using more advanced computational techniques would be necessary to spare time and resources. Therefore, pipelines that can integrate 1D sequence models for initial filtering of molecules that incorporate either 2D or 3D structural models for processing may offer the most comprehensive approach to drug discovery. In parallel, ESM-style protein language models contribute transfer-learned, self-supervised embeddings that improve GPCR tasks without labels, while equivariant and diffusion pose predictors such as EquiBind and DiffDock give rapid predictions on ligand poses which can be used to integrate with GPCRdb structures for multimodal training and rapid screening (Lin et al., 2023; Stärk et al., 2022; Corso et al., 2022). The most beneficial outcome for GPCR binding affinity predictions would be the success of generative AI; having the ability to use all dimensional models coherently to predict binding affinity accurately and at a rapid pace would outperform all current methods used for drug discovery. When combined with rigorous data standards and an explainable AI evaluation, self-supervised pretraining, along with generative diffusion, offers a credible pathway to high-throughput GPCR discovery.

## Strengths and limitations

GPCRs pose substantial challenges for models to address when predicting binding affinity. Receptors cycle through multiple conformational and signaling states, such as G-protein and β-arrestin pathways, giving rise to biased agonism; a single static structure cannot capture these features (Latorraca et al., 2017; Preininger et al., 2013). ML models often struggle to new GPCR subtypes when training is limited or imbalanced. If receptor structural data (crystal structures or AlphaFold models) are not incorporated, ML models miss receptor-specific features, limiting their ability to distinguish closely related subtypes. Subtle sequence and pocket differences across closely related GPCRs further influence bias through pocket shape, water networks, and side-chain rotamers, underscoring the need for approaches that can integrate dynamics with the limitations of experimental workflows (Venkatakrishnan et al., 2013; Michino et al., 2025). The influence of Ki, Kd, and IC50 values, with Ki and Kd being equilibrium affinity constants, whereas IC50 is a readout that depends on the experimental assay conditions; mixing them without careful normalization can introduce label bias (Gilson and Zhou, 2007; Kitchen et al., 2004). Thus, it is important to recognize the potential strengths and weaknesses of 1D, 2D, and 3D models pertaining to the GPCRs binding affinity prediction, as shown in Table 2.

1D models would do best for rapid screening of very large libraries (Wang, 2024). However, a potential issue for 1D-type models could be the impact of SMILES on the dataset used. Although it has been shown that CNNs using 1D inputs perform well under random splits, they collapse whenever there are unseen inhibitors, indicating that redundancy and leakage drive performance rather than learned interactions (Ong et al., 2023). When known SMILES are replaced with junk SMILES per inhibitor, accuracy remains unchanged and sometimes improves (Ong et al., 2023). This shows that these models mainly learn SMILES substrings as identifiers rather than structurally relevant features. This exposes a core limitation of SMILES encodings, where models can potentially fail to recognize that two different encodings can describe the same molecule, providing the need for more improved structure-based representations (Ong et al., 2023).

2D graph models are preferable for chemotype refinement when activity is driven by molecular topology (Liao et al., 2022). The trade-offs are that 2D encodings ignore stereochemistry and 3D orientations, which leads to dependence on approximations that can impact accuracy due to the sensitivity to input quality; this is especially true for the dynamic GPCR pockets (Wang, 2024; Congreve et al., 2020). Dataset biases could lead to inflated false-positive results; this emphasizes the need for bias-aware splits and validation (Ong et al., 2023).

3D models provide mechanisms that can be based on a specific active site, are preferred for subtype selectivity, allostery, and for determining how a ligand resides within a receptor which emphasizes their use when reliable, high-quality structures exist or when optimizing for selectivity and allosteric effects. (Gilson and Zhou, 2007; Spassov, 2024; Congreve et al., 2020; Kitchen et al., 2004; Cheng et al., 2012; Stärk et al., 2022; Corso et al., 2022). Despite the rapid progress, 3D GPCR binding-prediction needs more data for better efficacy; only a fraction of the ∼800 human GPCRs have been experimentally determined, which limits model training, pocket generalization, and the ability to study less-known receptors (Michino et al., 2025).

## Conclusion and future directions

Binding affinity prediction for GPCRs has progressed from fast but superficial 1D sequence models to structurally informed 2D graphs and fully 3D, structurally-based models. Each methodology offers strengths that could support the other. 1D models enable rapid screening of large libraries, 2D models enhance chemical context and substructure awareness, and 3D models can effectively capture pocket geometry, receptor states, and substrate selectivity. Robust pipelines for GPCRs would benefit from the combination of dimensional scales rather than choosing among them. The use of 1D sequence-based models for initial screening, 2D graph and attention architectures for chemotype refinement, and 3D structure-based models that focus on specific pockets and receptor states. When supported by high-quality data curation, consistent affinity measurements, and processes that account for bias, multidimensional workflows can yield more realistic generalizations with trustworthy results. Multimodal learning that aligns protein language model embeddings, pocket geometry, receptor state, and readouts related to signaling bias can improve the robustness of new data. Generative models that can propose ligands based on GPCR sequences, a set of different 3D pocket conformations, and desired biological profiles could bridge the gap between affinity prediction and *de novo* design. To make these systems optimal for clinical use, future work should prioritize GPCR benchmarks built on curated resources, utilizing scaffold and time-split evaluations, and incorporating explainable AI analyses that can link the model to relevant chemical and structural features. Ultimately, the most impactful GPCR discovery platforms will treat 1D, 2D, and 3D representations as complementary parts of the same system, where they can be integrated into reproducible workflows that support experimental design, explain failures, and accelerate the progression from virtual candidates to safe and effective drugs.

## Glossary

CI: Concordance Index (Calculates how well the model preserves the rank ordering of valid values)
RMSE: Root Mean Squared Error (Calculates the average magnitude of prediction errors, with larger errors penalized more)
**R** ^**2**^: Coefficient of Determination (Calculates the share of variance in the target that the model explains)
AUC: Area Under the ROC Curve (Calculates the probability that a random positive score will be higher than a random negative for binary classifications)
